# The oxygen isotope enrichment of leaf-exported assimilates – does it always reflect lamina leaf water enrichment?

**DOI:** 10.1111/nph.12359

**Published:** 2013-06-13

**Authors:** Arthur Gessler, Elke Brandes, Claudia Keitel, Sonja Boda, Zachary E Kayler, André Granier, Margaret Barbour, Graham D Farquhar, Kerstin Treydte

**Affiliations:** 1 Leibniz Centre for Agricultural Landscape Research, Institute for Landscape BiogeochemistryEberswalderstr. 84, 15374, Müncheberg, Germany; 2 INRA, UMR 1137 Ecologie et Ecophysiologie Forestières INRA/Université de Lorraine54280, Champenoux, France; 3 Research School of Biology, Australian National UniversityBuilding 46, Acton, ACT, 0200, Autralia; 4 Faculty of Agriculture and Environment, University of SydneyPrivate Bag 4011, Narellan, NSW, 2567, Australia; 5 Swiss Federal Research Institute WSL, Research Unit Landscape DynamicsZürcherstrasse 111, CH-8903, Birmensdorf, Switzerland

**Keywords:** broadleaf, conifer, diel course, oxygen atom exchange, phloem transport

## Abstract

- The oxygen stable isotope composition of plant organic matter (OM) (particularly of wood and cellulose in the tree ring archive) is valuable in studies of plant–climate interaction, but there is a lack of information on the transfer of the isotope signal from the leaf to heterotrophic tissues.
- We studied the oxygen isotopic composition and its enrichment above source water of leaf water over diel courses in five tree species covering a broad range of life forms. We tracked the transfer of the isotopic signal to leaf water-soluble OM and further to phloem-transported OM.
- Observed leaf water evaporative enrichment was consistent with values predicted from mechanistic models taking into account nonsteady-state conditions. While leaf water-soluble OM showed the expected ^18^O enrichment in all species, phloem sugars were less enriched than expected from leaf water enrichment in Scots pine (*Pinus sylvestris*), European larch (*Larix decidua*) and Alpine ash (*Eucalyptus delegatensis*).
- Oxygen atom exchange with nonenriched water during phloem loading and transport, as well as a significant contribution of assimilates from bark photosynthesis, can explain these phloem ^18^O enrichment patterns. Our results indicate species-specific uncoupling between the leaf water and the OM oxygen isotope signal, which is important for the interpretation of tree ring data.

## Introduction

Plant physiological responses to climatic and weather conditions in the past and at present are critical to understanding the impact of global climate change on ecosystem performance and resilience in the future (McDowell *et al*., 2008; Sternberg, 2009). Evaporative enrichment of ^18^O (Δ^18^O) in leaf water and subsequent ^18^O enrichment of assimilates can be used to characterize environmental and physiological factors that control transpiration, based on established mechanistic models (Craig & Gordon, 1965; Dongmann *et al*., 1974; Farquhar & Cernusak, 2005). The oxygen isotopic composition of tree rings as archives has been used to explore long-term precipitation patterns (Treydte *et al*., 2006), seasonality of precipitation (Saurer *et al*., 2002), tree responses to variations in atmospheric water demand and soil water availability (Barbour *et al*., 2002; Roden *et al*., 2005) and intra-annual patterns of transpiration rates (Gessler *et al*., 2009a).

The oxygen isotopic composition (δ^18^O) in leaf water reflects both δ^18^O of source water and the evaporative conditions in the leaf–atmosphere continuum (Dongmann *et al*., 1974; Roden & Ehleringer, 1999b; Barbour *et al*., 2004). During transpiration, the water isotopologues containing the lighter ^16^O isotope escape from liquid into vapour faster and diffuse faster than the heavier ones, thereby enriching the water in ^18^O at the sites of evaporation. This evaporative enrichment of water is governed by the bidirectional exchange of water vapour between the leaf and the ambient air, and so is affected by the vapour pressure deficit of the air and the isotopic composition of the water vapour (Craig & Gordon, 1965).

Isotopic composition of water in the mesophyll cells of the leaf lamina is influenced by the evaporative enrichment at the sites of evaporation. Lamina leaf water consists of a mixture of nonenriched xylem (source) water and water from the sites of evaporation (Farquhar & Lloyd, 1993). Newly produced assimilates are assumed to carry the signature of the lamina leaf water at the time when they were produced with an equilibrium fractionation factor (ε_wc_) resulting in carbonyl oxygen being 25–28‰ more enriched than the lamina water (Sternberg & Deniro, 1983; Yakir & Deniro, 1990). Barbour & Farquhar (2000) assume that sucrose, as the main leaf exported sugar, is in full isotopic equilibrium with cytoplasmic water and thus should carry the isotope signature of mean lamina leaf water plus ε_wc_. The often cited value of 27‰ for ε_wc_ (Barbour & Farquhar, 2000; Cernusak *et al*., 2003) is an average value of the difference in the ^18^O isotopic composition between the cellulose of different aquatic plants and tunicates and the water in which they grew (DeNiro & Epstein, 1981). A comparable enrichment of leaf cellulose above leaf water was also found for different nonwoody terrestrial plants (DeNiro & Epstein, 1979; Helliker & Ehleringer, 2002; Cernusak *et al*., 2003). The situation is more complex in nonleaf tissues including tree rings, which can serve as long term, dateable, archives for isotopic information. Cellulose in tree rings is affected by both the isotopic composition of leaf water, and the sugars produced therein, and the isotopic composition of the xylem water, which equals source water (Roden *et al*., 2000). This is because some of the oxygen atoms of the phloem-originating sucrose are assumed to be exchanged in the trunk with nonenriched xylem water during cellulose synthesis (Sternberg *et al*., 1986; Farquhar *et al*., 1998; Roden & Ehleringer, 1999a; Barbour, 2007). When averaged over a large number of species, 42% of the organic oxygen seems to be exchanged (Cernusak *et al*., 2005). However, discussion continues as to whether this exchange rate varies over the growing season or over longer time periods (Gessler *et al*., 2009a), or if it is rather unaffected by environmental conditions (Sternberg & Ellsworth, 2011).

Only recently, it was assumed that organic compounds transported in the phloem might be subjected to seasonally variable exchange with source water resulting in a partial uncoupling of the leaf water signal already on its way to the tree ring (Offermann *et al*., 2011). In agreement with this finding, Barnard *et al*. (2007) observed an ^18^O depletion of phloem organic matter (OM) compared to the values expected from leaf water enrichment. Because the phloem sugars convey the leaf water isotopic signal to the tree rings, a deeper understanding of the processes and mechanisms affecting phloem δ^18^O is crucial for the interpretation of the oxygen isotope composition tree ring archive as well as for the quantification of oxygen atom exchange during cellulose synthesis.

Exchange between water and organic oxygen in carbonyl groups can occur under normal physiological conditions (Sternberg *et al*., 1986) but exchange does not occur for other oxygen-containing functional groups such as hydroxyl or carboxyl groups (Barbour, 2007). The type of compounds transported in the phloem (Van Bel & Gamalei, 1992; Van Bel, 1993; van Bel & Hess, 2008) might strongly affect the overall δ^18^O of phloem OM (c.f. Schmidt *et al*., 2001) and also the oxygen isotope composition of tree ring tissue.

Measurements of δ^18^O in phloem OM and a comparison with lamina leaf water, and recently assimilated and leaf-exported OM are scarce (for an overview see Gessler *et al*., 2009a), and hence we examined three different angiosperm and two gymnosperm tree species covering a broad range of tree life forms: (1) angiosperm, broadleaf, deciduous – sessile oak (*Quercus petraea*), European beech (*Fagus sylvatica*); (2) angiosperm, broadleaf, evergreen – Alpine ash *(Eucalyptus delegatensis*); (3) gymnosperm, coniferous, evergreen – Scots pine (*Pinus sylvestris*); (4) gymnosperm, coniferous, deciduous – European larch (*Larix decidua*). We carried out experiments over diel or diurnal courses to address the following questions: (1) Does the oxygen isotope enrichment of leaf sugars and phloem-transported assimilates generally reflect lamina leaf water enrichment in all species? (2) If not, can differences among species be attributed to the chemical composition of phloem-transported compounds or (3) are differences related to leaf/canopy morphological (e.g. needles vs broadleaves) or physiological traits? To answer these questions we measured and modelled (lamina) leaf water ^18^O enrichment over the diel course and related these values to water-soluble leaf OM (as a proxy for leaf sugars) and phloem-transported OM. In addition, we determined the phloem metabolome in oak, beech and pine to account for possible differences in the organic compounds metabolized between species and their contribution to the phloem ^18^O signature.

The questions we raise here are highly relevant to studies that use the δ^18^O of plant OM for physiological or climatic insights. δ^18^O of plant organic material can only provide an integrative tool to study the evaporative and photosynthetic processes that drive or are associated with plant water fluxes (Werner *et al*., 2012) if δ^18^O in phloem OM reflects the oxygen isotopic signature of leaf water or if changes in δ^18^O during phloem loading and/or transport can be accounted for. Moreover, if phloem OM δ^18^O does not directly reflect the leaf water oxygen isotope signature, interpretation of tree ring δ^18^O data would need to be reconsidered as cellulose is produced from the phloem-transported sugars.

## Materials and Methods

### Experimental sites

Experiments were performed at four different sites (Hartheim (*Pinus sylvestris* L.), Hesse II (*Fagus sylvatica* L. and *Quercus petraea* (Matt.) Liebl.), Tumbarumba (*Eucalyptus delegatensis* R. T. Baker) and Lötschental (*Larix decidua* Mill.)) throughout Europe and Australia. The main site characteristics are given in Table 1. Additional detailed information on the sites can by found in Holst *et al*. (2008; Hartheim), Granier *et al*. (2000; Hesse), Gessler *et al*. (2007a; Tumbarumba) and Moser *et al*. (2010; Lötschental).

**Table 1:** Main characteristics of the four experimental sites

Site	Location	Altitude (m asl)	Forest type	Target tree species	*T*_air_ (°C)	Precip. (mm)	Soil type	Tree height (m)	Tree age (yr)	LAI	Stand density (trees ha^−1^)
Hartheim	47°56′ N 7°37′ E Germany	201	Pine plantation	*Pinus sylvestris*	10.3	642	Calcaric Regosol	14.3	40	1.9	800
Hesse II	48°40′N 7°05′ E France	300	Mixed deciduous forest	*Fagus sylvatica Quercus petraea*	9.2	820	Luvisol/stagnic Luvisol	8.6	15–25	7.5	17820
Tumbarumba	35°39′ S, 148°09′ E Australia	1200	Wet sclerophyll forest	*Eucalyptus delegatensis*	5.0 (min) 18.7 (max)	1260	Acidic, eutrophic, red dermosol	40	Up to 90	1.4	nd
Lötschental^1^	46°23′N, 7°44′E Switzerland	2100 (treeline) 1350 (valley)	Alpine coniferous	*Larix decidua*			Podzolic Cambisols	20	150-200	< 2	nd

Target tree species: species measured and sampled for isotope analyses. In Hesse II two species were examined. *T*_air_, long-term mean annual air temperature; average daily temperatures are given for Hartheim and Hesse, average daily maximum (max) and minimum (min) temperatures are given for Tumbarumba. For this site *T*_air_ was measured at a 40 km distance from the flux station and at an elevation of 750 m. Precip, annual sum of precipitation; for Hartheim and Hesse long-term average sums are given, for Tumbarumba the average sum of the years 2002–2004 is shown. LAI, leaf area index; nd, not determined.

1 At the Lötschental site two stands located at two altitudes, one at the upper tree line of a south-facing slope and the other at the valley bottom on a rocky hill were examined. During the 2008 growing season, the mean temperature was 11.6°C at the valley bottom and 8.3°C at the treeline. Hydrological conditions are generally drier at the valley site due to lower precipitation (292 mm vs 410 mm; week 25–42).

### Meteorological measurements

Air temperature (*T*_a_), vapour pressure deficit (VPD) and photosynthetically active radiation (PAR) were determined above the canopy at the Hartheim, Hesse II and Tumbarumba sites. For details of the instrumentation see Holst *et al*. (2008), Granier *et al*. (2000) and Leuning *et al*. (2005), respectively. At the Lötschental sites, the meteorological parameters were determined 2 m above ground in open patches close to the trees examined. All data are averages over a 30-min period.

### Experimental set up and sampling of plant material

#### Hartheim

The measurement campaign (*P. sylvestris*) took place on 19.ix.2005 (first sampling: 10:00 h) and 20.ix.2005 (last sampling: 13:30 h) on three dominant trees growing close to a scaffold tower. Needles and twigs were sampled and gas exchange measurements performed in the sun-exposed part of the upper crown of the trees every 4 h (with the exception of the last sampling round which was started half an hour earlier). Needles were separated into current and previous year needles. We determined bulk leaf water ^18^O evaporative enrichment for both current and previous year needles separately once during the night (02:00 h) and once during the day (14:00 h). The measured values were not significantly different between the two needle age classes (*P *<* *0.05; *n *=* *3) at either time. As a consequence, both age classes were bulked for further analyses of the isotopic composition of leaf water and OM.

#### Hesse II

The measurements in Hesse were performed between 26.ix.2005 and 27.ix.2005. In total, eight sampling/measurement rounds were performed for both tree species (*F. sylvatica* and *Q. petraea*) starting at 11:30 h and terminating at 14:00 h on the following day. Using scaffolding, the leaves and twigs from the upper sun crown of three representative trees per species were harvested and at the same time gas exchange was determined.

#### Tumbarumba

The measurements were carried out between 9.iii.2005 and 10.iii.2005. Five measurement rounds were performed starting at 08:30 h and terminating at 08:00 h on the following day. We selected three representative dominant individuals of *E. delegatensis* with a maximum height of *c*. 38 m. The canopy of the trees was accessed for leaf and twig sampling as well as for gas exchange measurements using a 40-m hydraulic lift installed on a truck.

#### Lötschental

At both sites (valley and treeline) we selected four representative *L. decidua* trees. The measurement campaigns at the valley bottom were performed on 10.vi.2008 and 14.vii.2008. At the treeline one campaign was carried out on 18.viii.2008. The stands were quite open, so that even the lowest branches (which could be accessed from the ground) of all individuals were exposed to direct sunlight. From these lower branches needles and twigs were harvested seven times during the light period starting at 06:00 h in June and at 07:00 h at the two other dates. From the twigs we collected phloem exudates as well as xylem water.

### Extraction of leaf water, xylem water and OM fractions

#### Bulk leaf water

Needles of the current and recent year flush (*P. sylvestris*), current year needles (*L. decidua*) and leaves (*F. sylvatica*, *Q. petraea* and *E. delegatensis*) were quickly transferred into airtight closed glass tubes and frozen. Needle/leaf water was obtained by cryogenic vacuum extraction according to Ehleringer *et al*. (2000).

#### Xylem water

Twig xylem water of pine, oak, beech and Alpine ash was extracted from cut twigs by applying a gentle vacuum following Brandes *et al*. (2007). For larch the xylem water was extracted from the woody tissue of twigs by cryogenic vacuum extraction. Brandes *et al*. (2007) showed that both methods result in comparable values for xylem water δ^18^O.

#### Phloem exudates

For all five species, bark pieces removed from the twigs were placed into 1.8-ml deionized water volumes according to the procedure described by Gessler *et al*. (2004) avoiding contamination of the phloem exudates by cellular constituents (Schneider *et al*., 1996). We refer to the OM fraction obtained with this exudation procedure described above as bulk phloem OM. In a further step the exudates of pine, oak and beech were passed through Dowex-50 (H^+^) and Dowex-1 (Cl^−^) columns in series to separate neutral sugars from amino acids and organic acids as described by Richter *et al*. (2009). The eluate collected is referred to as neutral phloem sugars.

For beech and pine we tested whether the exchange of oxygen atoms occurred between bulk phloem OM and the exudation solution as done previously by Offermann *et al*. (2011). For this purpose pairs of bark pieces were harvested in the direct vicinity of a given twig. One of these bark samples was incubated in relatively ^18^O depleted deionized water (δ^18^O: −8.9 ± 0.3‰) and the other one in relatively enriched water (δ^18^O: 8.9 ± 2.5‰). The oxygen isotope signatures of bulk phloem OM did not differ between the two exudation solutions (beech: δ^18^O of bulk phloem OM exuded in enriched water: 28.5 ± 1.1‰ and in depleted water: 28.4 ± 0.9‰ (*n *=* *11); pine: δ^18^O in enriched water: 30.1 ± 0.8‰ and in depleted water: 30.3 ± 1.2‰ (*n *=* *10)). We thus can exclude any oxygen atom exchange during the exudation procedure.

#### Leaf water-soluble OM

For all five species water-soluble OM was extracted from the leaves and needles according to a modification of the method described by Gessler *et al*. (2009b). Frozen leaf material was denatured in a microwave oven for 1 min and dried at 50–60°C. Leaves were ground with a ball mill (MM 200; Retsch GmbH, Haan, Germany). Aliquots of 50–70 mg were extracted in 1.5 ml deionized water on a shaker for 1 h at 4°C and subsequently put in a water bath at 95°C for 1 min for denaturation of soluble proteins. After cooling on ice, samples were centrifuged at 4°C and 12 000 ***g*** for 10 min. Supernatants were stored at −20°C until further analysis.

### Collection of water vapour

At the Hartheim, Hesse II and Tumbarumba sites atmospheric water vapour was collected during the day between 13:00 and 14:00 h and during the night between 02:00 and 03:00 h by cryogenic condensation from middle canopy height. The air was pumped through tubing at a flow rate of *c*. 1 l min^−1^ and through a glass u-tube inserted in a dewar containing an ethanol – dry ice slush at −70 to −80°C. Water vapour was collected in a cold trap for 1–2 h and kept frozen until δ^18^O analysis. We averaged the daytime and night-time values, giving mean values of −17.9‰, −15.2‰ and −17.6‰ for Hartheim, Hesse II and Tumbarumba, respectively.

### Gas exchange measurements

Leaf gas exchange of beech, oak, Alpine ash and pine was measured in the upper sun-exposed canopy in the direct vicinity of the twigs sampled for isotope analysis. For the three broadleaf species three leaves were measured per individual tree with a broadleaf chamber connected to a portable gas exchange system (LI-COR 6400: Li-Cor Biosciences, Lincoln, NE, USA; or GFS3000: Heinz Walz GmbH, Effeltrich, Germany). For pine, small twigs (with their needles attached) were inserted into a conifer leaf chamber attached to the same gas exchange system. All measurements were conducted under ambient light and temperature conditions. Pine twigs with previous and current year needles attached were placed in the gas exchange chamber. In accordance with the results of Beadle *et al*. (1985) we assumed gas exchange and stomatal conductance of the age classes to be equal at that time of the year. For all four species net CO_2_ and H_2_O exchange rates were measured, and stomatal conductance (*g*_s_) was subsequently calculated according to von Caemmerer & Farquhar (1981). The temperature of beech and oak leaves was determined with a thermocouple; for pine needles the temperature was assumed to be equal to ambient air temperature as suggested by Walcroft *et al*. (1997), because needles are thought to be strongly coupled with the environment. The leaf area of the needles inserted into the chamber was determined according to Barnard *et al*. (2007).

### Isotope measurements

The determination of δ^18^O in water and OM samples was performed using a TC/EA high temperature conversion/elemental analyser coupled to either a DeltaPlus XP (ThermoFisher, Bremen, Germany) or an Isoprime (Micromass, Middlewich, UK) mass spectrometer. The precision was < 0.17‰. The oxygen stable isotope composition is expressed using small delta notation in parts per thousand, relative to the international Vienna Standard Mean Ocean Water (VSMOW) standard.

#### Bulk leaf water, cryogenically trapped water vapour and xylem water

Water samples of 0.7 μl were injected into the TC/EA. Each sample was analysed four times in sequence and only the value of the last measurement was taken into account to avoid memory effects.

#### Phloem bulk OM, phloem neutral sugars and leaf water-soluble OM

Samples of 40–100 μl of the phloem exudation solution or of the leaf extracts were transferred to silver capsules and water was evaporated at 60°C in an oven. The samples contained on average *c*. 300 μg organic O.

### Isotopic calculation and modelling

The oxygen isotope enrichment of bulk leaf water or plant OM (leaf water-soluble OM, bulk phloem OM, neutral phloem sugars) is expressed as enrichment above source water (Δ^*18*^O):


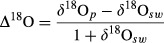
Eqn 1

(δ^18^O_*p*_, oxygen isotopic composition of leaf water or plant OM; δ^18^O_*sw*_, isotopic composition of the source water). In our case we calculated the ^18^O enrichment based on xylem water as source water.

According to Farquhar & Gan (2003), the ^18^O enrichment of bulk leaf water is given by:


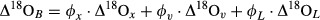
Eqn 2

(φ_*x*_, φ_*v*_ and φ_L_, proportions of total water associated with the longitudinal xylem, the veinlets and the lamina mesophyll, respectively; Δ^18^O_*x*_, Δ^18^O_*v*_ and Δ^18^O_L_, evaporative enrichment of xylem, veinlet and lamina leaf water above source water, respectively). Lamina leaf water is the reaction water in which assimilates are produced, and thus it is necessary to estimate Δ^18^O_L_ from measured Δ^18^O_*B*_. Following Farquhar & Gan (2003), we assumed the water volume in the veinlets to be negligible. Because we excluded the main veins from the leaf sample of beech and oak, we assumed for these two species that mesophyll water composition was equivalent to the isotopic composition of extracted bulk leaf water. For the coniferous species and for Alpine ash we applied the procedure described in detail by Barnard *et al*. (2007) to estimate φ_*x*_. Lin *et al*. (2001) showed that the contribution of xylem to the cross-sectional area of current year Scots pine needles (sampled in October) was 2.2%. A comparable xylem contribution of 3% has been observed for larch (Takemoto & Greenwood, 1993). From cross-sectional cuttings of leaf samples from *E. delegatensis* we estimated the main vein to contribute to 10% of the leaf water volume. From these xylem areas, φ_*x*_ values were calculated following Barnard *et al*. (2007) and Δ^18^O_L_ was estimated from Δ^18^O_*B*_. For needles we assumed leaf vein xylem water not to be enriched in ^18^O, for *E. delegatensis* we determined major vein water to be enriched by 2.8‰ above source water.

Leaf water enrichment during measurement campaigns was modelled with approaches of increasing complexity as described in detail by Barnard *et al*. (2007) for pine, oak, beech and Alpine ash. We calculated the isotopic enrichment of ^18^O over source water at the site of evaporation in the leaf (Δ^18^O_*es*_) under steady-state conditions and lamina leaf water enrichment under steady-state (Δ^18^O_Ls_) and nonsteady-state conditions (Δ^18^O_Ln_). Details are given in the Supporting Information Notes S1.

For pine and Alpine ash we additionally modelled Δ^18^O_Ls_ for the 2 d before gas exchange measurements and leaf and phloem sampling were performed. For these 2 d we calculated stomatal conductance and transpiration based on their relationship with VPD determined during the measurement campaigns. Leaf temperature was assumed to equal air temperature and the scaled effective path length for water movement from the xylem to the site of evaporation (L) was assumed not to be different from the value determined during the measurement period.

In order to calculate mean daytime oxygen isotope enrichment of leaf water and phloem OM above twig xylem water (weighted Δ^18^O) for pine, oak, beech and Alpine ash, the Δ^18^O values (see Eqn 1) of each daytime measurement time were weighted by the corresponding CO_2_ assimilation rate measured (A, mol m^−2^ s^−1^), according to Cernusak *et al*. (2005):


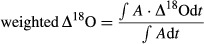
Eqn 3

(numerator, daily (daytime) integral of the product of *A* and Δ^18^O (‰ mol m^−2^); denominator, daily integral of *A* (mol m^−2^)). No gas exchange measurements were performed with larch, so we weighted measured leaf water Δ^18^O values by the half-hourly averages of photosynthetic active radiation before harvest as a rough proxy for photosynthesis. We also weighted modelled Δ^18^O_Ls_ of pine and ash from the 2 d preceding the gas exchange measurements. To obtain *A* values for this time period (when no gas exchange measurements were performed) we calculated a photosynthesis–light response curve for the gas exchange measurement period and calculated *A* from photosynthetically active radiation.

### Phloem metabolite analysis

Samples (250 μl) of the bulk phloem exudate of pine, oak and beech were dried under vacuum and derivatisation (methoxyamine and silylation with N-methyl-N-trimethylsilylfluoroacetamide) as well as GC-MS analysis for metabolite profiling were performed without further purification according to Erxleben *et al*. (2011). The derivatised phloem samples were injected into a GC-quadrupole MS system (GC: 7890A; MS: 5975C; Agilent Technologies, Waldbronn, Germany) operating in electron impact ionization. Data were deconvoluted and peak areas quantified using the AMDIS software (http://chemdata.nist.gov/mass-spc/amdis/). Peak identifications were carried out by matching retention indices and mass spectral similarity against the NIST 05 mass spectral library (http://www.nist.gov/srd/nist1a.cfm) and a user-defined metabolite library based on the Golm metabolome database ((Kopka *et al*., 2005); http://csbdb.mpimp-golm.mpg.de/csbdb/gmd/msri/gmd_msri.html). For the 25 most abundant identified compounds calibration curves were made with external standards to quantify metabolite concentrations.

### Statistical analyses

All statistical analyses were performed using NCSS 2004 (Number Cruncher Statistical Software, Kaysville, UT, USA). Differences in ε_wc_ between different OM pools were determined using analysis of variance (GLM-ANOVA). Regression lines between Δ^18^O values of leaf and phloem OM pools were determined by linear regression analysis.

## Results

### Environmental parameters at the field sites

**Figure 1:**
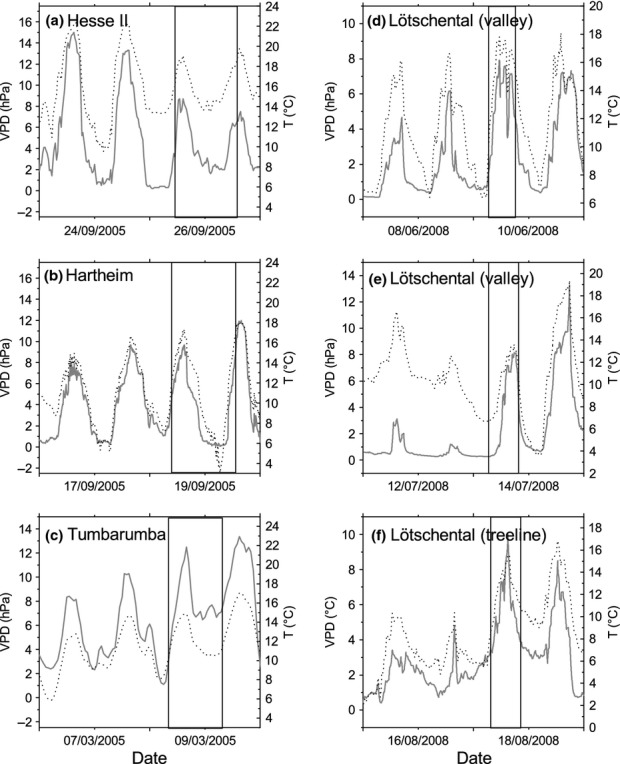
Water vapour pressure deficit of the ambient air (VPD, grey bold line) and air temperature (T, black dotted line) at Hesse II (beech (*Fagus sylvatica*) and oak (*Quercus petraea*)) (a), Hartheim (pine (*Pinus sylvestris*)) (b), Tumbarumba (Alpine ash (*Eucalyptus delegatensis*)) (c), and Lötschental (larch (*Larix decidua*)) (d–f) before and during the experimental period. (d) and (e) indicate the June and July sampling periods in the valley site and (f) the sampling period at the treeline site in Lötschental. The frames indicate the time periods of sampling and measurement

In Hesse II, the highest air temperature during the measurement campaign was 19.1°C (Fig. 1a). VPD varied between 1.6 and 8.7 hPa. Maximum daytime temperatures were 22°C during the 2 d before the campaign and were thus higher than during the measurements. Day-to-night differences in VPD and maximum VPD values were also greater on the days preceding the sampling. At the Hartheim site (Fig. 1b) the maximum air temperature during the measurement campaign was 17.3°C. VPD ranged from 0.1 to 9.7 hPa during that period. VPD and air temperature values were comparable during the 2 d preceding the measurement campaign with the exception of higher minimum night-time temperatures between 17 and 19.ix.2005. At Tumbarumba the highest air temperature during the measurement campaign was 14.8°C and VPD reached 12.5 hPa (Fig. 1c). Somewhat lower maximum and minimum VPD and air temperature values were observed during the 2 d before the campaign. At the Lötschental valley site, the temperature range during the June measurement campaign was between 6.4 and 17.9°C and VPD reached a maximum of 9.2 and a minimum of 0.9 hPa (Fig. 1d). Both maximum temperatures and maximum VPD were slightly lower during the preceding 2 d, whereas minimum values of both, temperature and VPD were comparable among diel courses. During the July campaign at the Lötschental valley site the VPD range was 0.3–8.1 hPa and the temperature range 6.8–13.6°C (Fig. 1e). The maximum air temperature was comparable on the preceding day but rose to 16.6°C 2 d before the campaign. The maximum VPD was 3.2 and 1.2 hPa on 12 and 13.vii.2008, respectively. At the Lötschental treeline site VPD varied between 3.4 and 10.0 hPa on the measurement day (Fig 1f). The minimum temperature during sampling was 8.4°C and a maximum of 15.8°C was reached in the afternoon. Both temperature and VPD were lower during the two preceding days, reaching maximum values of 4.8 hPa and 10.4°C, respectively.

### Leaf water evaporative enrichment - observed and modelled values

**Figure 2:**
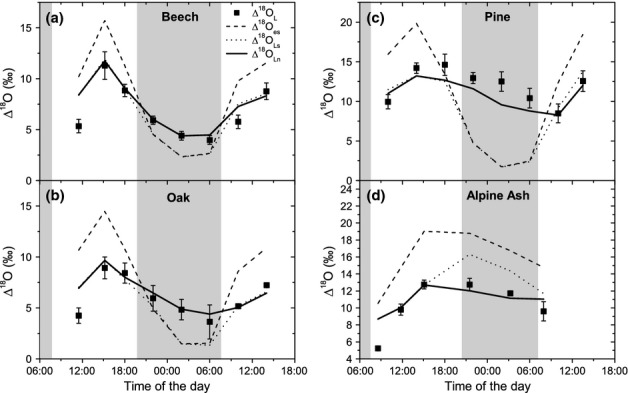
Evaporative enrichment of leaf water. Comparison between the diel course of oxygen isotopic enrichment (Δ^18^O) for beech (*Fagus sylvatica*) (a), oak (*Quercus petraea*) (b), pine (*Pinus sylvestris*) (c) and Alpine ash (*Eucalyptus delegatensis*) (d) as observed in the lamina leaf water (Δ^18^O_L_) and the values estimated from models for isotopic enrichment at the site of evaporation (Craig and Gordon model, Δ^18^O_es_) and for mean lamina leaf water: Δ^18^O_Ls_ and Δ^18^O_Ln_, Péclet-based steady-state and nonsteady-state models, respectively. Vertical error bars indicate the standard error of the mean of three plants. White/shadowed areas denote light/dark periods. The observed values are means from three replicates (i.e. three individual trees) ± SD.

In beech (Fig. 2a) and oak (Fig. 2b) leaf water ^18^O enrichment (Δ^18^O_L_) showed comparable diel courses with maximum values after midday and a minimum before sunrise at 06:00 h. The observed range of Δ^18^O_L_ was between 4.0‰ and 11.3‰ for beech and between 3.6‰ and 8.9‰ for oak. This enrichment was slightly higher with pine (Fig. 2c). The diel pattern was time-shifted in pine, with the maximum Δ^18^O_L_ being observed in the late afternoon at 18:00 h and the minimum after sunrise at 10:00 h. In Alpine ash, the highest leaf water ^18^O enrichment was observed in the afternoon and during the first part of the night (Fig. 2d). For all four species, daytime values of measured Δ^18^O_L_ were consistently lower than calculated Δ^18^O_es_. The scaled effective path length (L) that was used to calculate the Péclet number (Eqn S2) was estimated based on the discrepancy between the predicted Δ^18^O_es_ and observed Δ^18^O_L_ at time points between 13:30 and 18:00 h, when Δ^18^O_L_ was assumed to be at steady state. We calculated an L of 0.017, 0.021, 0.060 and 0.025 m for beech, pine, oak and mountain ash, respectively. While the predictions of mean lamina leaf water enrichment that assumed isotopic steady state (Δ^18^O_Ls_) were in rather good agreement with the observed enrichment during the light period, they underestimated Δ^18^O_L_ in beech, oak and pine during the night. In Alpine ash, both calculated Δ^18^O_es_ and Δ^18^O_Ls_ were higher than the observed values during the night, mainly due to the high night-time VPD during the measurement campaign (cf. Fig. 1c) The nonsteady-state model (Δ^18^O_Ln_) finally predicted lamina leaf water evaporative enrichment adequately for the whole diel course in all four species (Fig. 2) with only a slight underestimation of night-time values for pine.

### Transfer of leaf water ^18^O enrichment to recent assimilates

**Figure 3:**
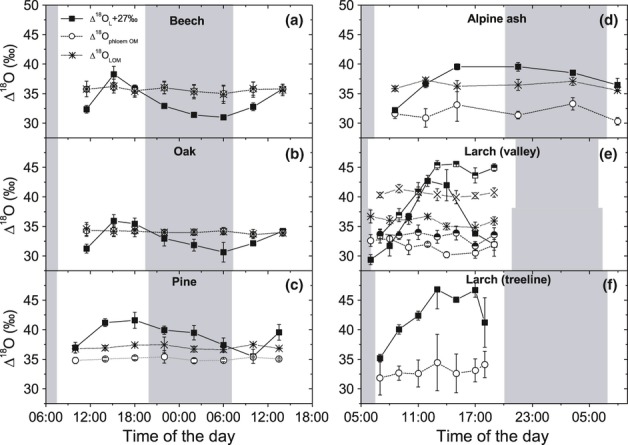
Comparison between ^18^O enrichment of bulk phloem organic matter (Δ^18^O_phloem_), leaf water-soluble organic matter (Δ^18^O_LOM_) and leaf water during the diel or diurnal course for beech (*Fagus sylvatica*) (a), oak (*Quercus petraea*) (b), pine (*Pinus sylvestris*) (c) Alpine ash (*Eucalyptus delegatensis*) (d) and larch (*Larix decidua*) (e,f). For a better comparison of the absolute values we have displayed leaf water enrichment + ε_wc_ (Δ^18^O_L_ + 27‰). In (e) the measurements in the valley in June and July are shown; here the June values are shown with regular symbols and the July values with half-filled symbols for phloem OM and leaf water and with a cross for leaf water-soluble OM. (f) Data for larch from the treeline. White/shaded areas denote light/dark periods; in (e) the upper half indicates the light/dark situation in June, the lower half in July. Data shown are mean values ± SD (*n *=* *3–4).

Newly produced assimilates carry the signature of the leaf water at the time when they are produced with an equilibrium fractionation factor (ε_wc_) resulting in the carbonyl oxygen being *c*. 27‰ more enriched than leaf water (Sternberg & DeNiro, 1983; Yakir & DeNiro, 1990). As a consequence, we compared Δ^18^O_L_ + 27‰ with Δ^18^O of bulk phloem and leaf water-soluble OM during the diel/diurnal course for beech (Fig. 3a), oak (Fig. 3b), pine (Fig. 3c), Alpine ash (Fig. 3d) and larch (Fig. 3e,f). For all five species it was obvious that the maximum variations in Δ^18^O of phloem OM (beech, 1.2‰; oak, 0.7‰; pine, 0.6‰; Alpine ash, 2.9‰; larch, 2.7‰) and of leaf water-soluble OM, which has a comparable range of variation, were much smaller than those of leaf water (beech, 7.3‰; oak, 5.3‰; pine, 6.1‰; Alpine ash, 7.4‰; larch, 13.4‰).

In the two broadleaf deciduous species, the Δ^18^O of bulk phloem OM was lower than or similar to Δ^18^O_L_ + 27‰ at midday and in the afternoon and higher in the dark period and in the morning. In pine and Alpine ash, bulk phloem OM Δ^18^O was below Δ^18^O_L_ + 27‰ during the whole diel course. For larch, where we only carried out measurements during the light period, bulk phloem OM ^18^O enrichment was higher than Δ^18^O_L_ + 27‰ at the valley bottom in June and July (Fig. 3e) until 08:00 h in the morning, but fell below Δ^18^O_L_ + 27‰ by up to 12.1‰ during the rest of the light period. At the tree line in August, Δ^18^O of bulk phloem OM of larch was lower than Δ^18^O_L_ + 27‰ during the whole daytime measurement period (Fig. 3f). The average diel (day and night) bulk phloem Δ^18^O was 35.7, 34.1, 35.1, 31.2‰ in beech, oak, pine and Alpine ash, respectively. The average daily bulk phloem Δ^18^O in larch was between 31.7 and 33.4‰ depending on time point and site. Clearly Δ^18^O of leaf soluble OM was higher than that of bulk phloem OM in pine, Alpine ash and larch.

Photosynthesis-weighted daytime Δ^18^O of lamina leaf water was compared with the diel nonweighted or daytime-photosynthesis-weighted average of Δ^18^O of phloem OM for beech, oak, pine and Alpine ash (Table 2). For larch, the Δ^18^O of leaf water was weighted by photosynthetic active radiation as a proxy. In the two broadleaf deciduous species the difference between weighted Δ^18^O_L_ and Δ^18^O phloem OM amounted to *c*. 27‰, independent of the averaging and weighting procedure for the phloem values. However, the difference for the two coniferous species and the evergreen broadleaf species was lower than 27‰, reaching values as low as 16.7‰ for larch trees growing at the tree line. The data from pine and Alpine ash show that these values were consistently low, independent of whether only daytime or day-plus-night averages for Δ^18^O of bulk phloem OM were used for the comparison. There was no difference in the ^18^O isotopic signature between phloem bulk OM and the phloem neutral OM fraction for the three species (beech, oak, pine) examined.

**Table 2:** Difference between leaf water evaporative enrichment and Δ^18^O in fast-turn over organic matter (OM) pools (ε_wc_) in leaf and phloem

Species	Beech (*Fagus sylvatica*)	Oak (*Quercus petraea)*	Pine (*Pinus sylvestris*)	Alpine ash (*Eucalyptus delegatensis*)	Larch (*Larix decidua*)							
Site	Hesse II	Hesse II	Hartheim	Tumbarumba	Lötschental (valley)		Lötschental (treeline)
Month	September	September	September	March	June	July	August
Leaf water-soluble OM	27.2 ± 0.4a	26.7 ± 0.6a	25.5 ± 0.6a	26.8 ± 0.9a	26.3 ± 0.4a	25.7 ± 0.5a	nd
Phloem bulk OM	27.0 ± 0.5a	26.7 ± 0.5a	23.5 ± 0.6b	22.2 ± 0.8b	22.0 ± 0.6b	18.5 ± 1.1b	16.7 ± 1.0
Phloem bulk OM^*^	27.2 ± 0.5a	26.7 ± 0.6a	23.5 ± 0.7b	22.2 ± 1.0b			
Phloem neutral fraction	27.1 ± 0.4a	26.7 ± 0.5a	23.4 ± 0.7b	nd	nd	nd	nd

Observed daytime leaf water enrichment (‰) was weighted for photosynthesis (beech, oak, pine and Alpine ash) or photosynthetic active radiation (larch) and related to the nonweighted diel average of the OM pools with the exception of Phloem bulk OM^*^, where phloem OM values during the light period were also weighted for photosynthesis. Data shown are average values from three to four trees per species ± SD. Different letters indicate significant differences among the different OM fractions in a given species (at a given site/time point). nd, not determined.

We might assume that turnover times for phloem OM are longer than those for leaf water and that consequently Δ^18^O of phloem OM might be influenced by leaf water enrichment from preceding days. Accordingly, we calculated Δ^18^O_Ls_ of pine and Alpine Ash for the light period (where it showed quite good agreement with measured leaf water values; cf. Fig. 2) for the 2 d before phloem sampling. For pine, photosynthesis-weighted Δ^18^O_Ls_ for the preceding 2 d was estimated to be 12.8‰ on average and was thus slightly higher than the weighted observed Δ^18^O_L_ (11.4‰) during the sampling period. The difference between Δ^18^O_Ls_ from the two preceding days and the average Δ^18^O phloem OM during the sampling period amounted to 22.3‰, thus deviating by 4.7‰ from the theoretical values of 27‰. For Alpine ash, the calculated weighted Δ^18^O_Ls_ of the two preceding days was comparable (9.3‰) with measured Δ^18^O_L_ (8.9‰) during the campaign. Thus, the difference between this modelled Δ^18^O_Ls_ and the measured Δ^18^O phloem OM was still clearly lower than the expected 27‰.

**Figure 4:**
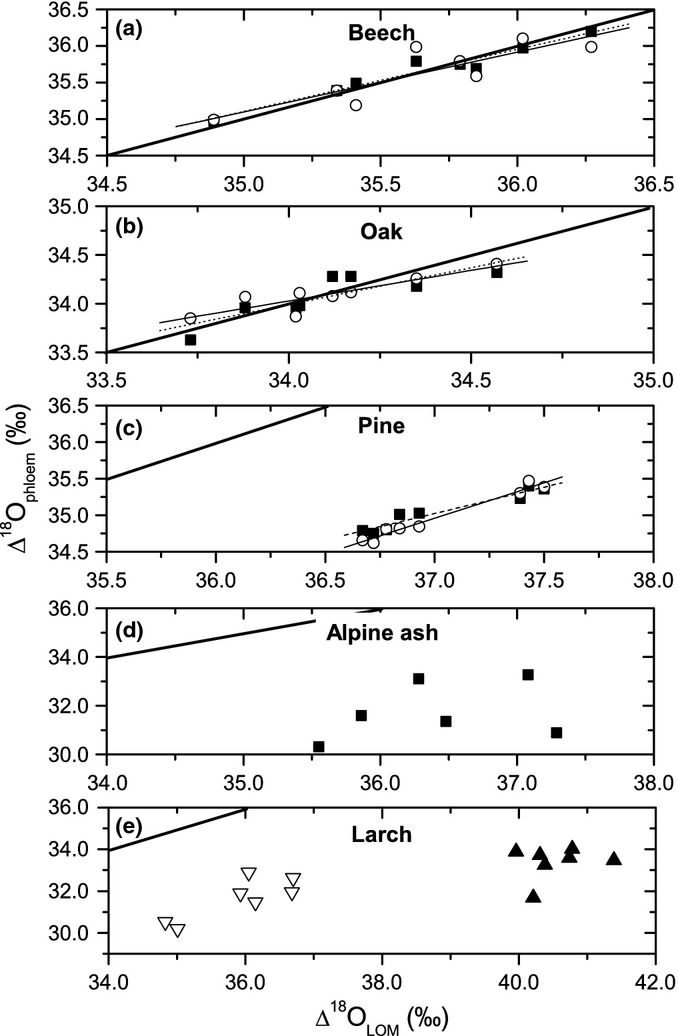
Relationship between ^18^O enrichment of water-soluble leaf organic matter (Δ^18^O_LOM_) as a proxy for leaf sugars and phloem organic matter (Δ^18^O_phloem_) for beech (*Fagus sylvatica*) (a), oak (*Quercus petraea*) (b) pine (*Pinus sylvestris*) (c) Alpine ash (*Eucalyptus delegatensis*) (d) and larch (*Larix decidua*) (e) during the diel or diurnal course. In (a–c) two different phloem OM fractions are shown: black squares (and dotted regression lines) refer to bulk phloem exudates; white circles (and solid regression lines) to the neutral OM fraction. *R*^2^ for the regression lines are between 0.71 and 0.96. In (e) only bulk phloem OM and in (d) data for bulk phloem exudates from two time points are shown: open reverse triangles, values from June; black triangles, from July. All data shown are mean values from three to four replicates (i.e. individual trees). The average standard deviation of the mean values shown over all species is 1.1‰ for ^18^O enrichment of phloem exudates and 0.8 ‰ for leaf water-soluble OM.

The comparison between values of leaf water-soluble OM and bulk phloem OM for all five species, and the comparison between Δ^18^O values of leaf water-soluble OM and the Δ^18^O of neutral phloem sugars for beech, oak and pine, are illustrated in Fig. 4. For the two broadleaf deciduous species (Fig. 4a,b), the Δ^18^O values of both bulk phloem OM and neutral phloem sugars were similar to leaf water-soluble OM during the whole diel course, as indicated by the points scattering around the 1 : 1 line. In contrast to beech and oak, the two phloem fractions in pine were both depleted on average by 2.0‰ compared to leaf water-soluble OM (Fig. 4c). For Alpine ash the bulk phloem fraction was on average depleted by 4.7‰ and for larch by 4.3‰ (June) and 7.2‰ (July) compared to leaf water-soluble OM.

### Phloem metabolic profile

**Figure 5:**
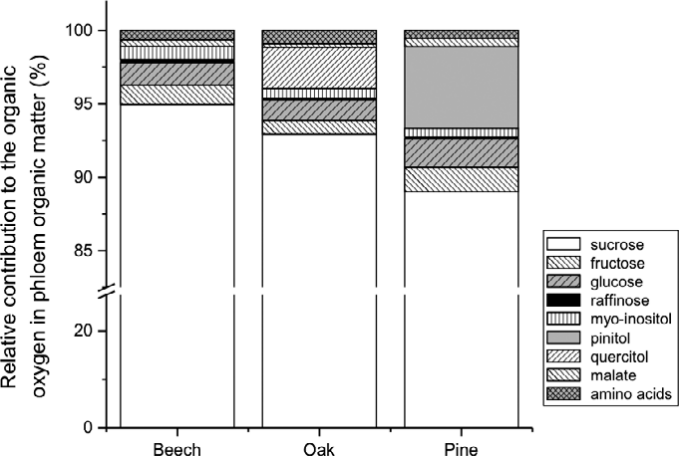
Relative contribution of different organic compounds to the organic oxygen in bulk phloem exudates in beech (*Fagus sylvatica*), oak (*Quercus petraea*) and pine (*Pinus sylvestris*). Data shown are mean values from three trees of each species. Phloem exudates were collected between 13:00 and 14:00 h.

Different metabolites might have different ^18^O isotopic compositions and thus might be a source of variation in Δ^18^O phloem OM among species. In all three species examined sucrose contributed most to the organic oxygen in phloem OM (Fig. 5) making up 95% (beech), 93% (oak) or 89% (pine). Fructose and glucose together made up between 2.3% and 3.6% and raffinose, myo-inositol, malate and the sum of amino acids each contributed < 1% in each species. Cyclitols were present in the bulk phloem OM of oak and pine: Quercitol contributed 2.8% to organic oxygen in phloem OM in oak and pinitol 5.6% in pine.

## Discussion

### Observed lamina leaf water Δ^18^O is consistent with values predicted from mechanistic models

The simplest evaporative enrichment model (Eqn S1) considers enrichment at the sites of evaporation only (Δ^18^O_es_). The second model (Eqn S2) adds a Péclet effect (Δ^18^O_Ls_) and the final model (Eqn S3) also takes into account leaf isotopic nonsteady-state conditions (Δ^18^O_Ln_). In all four species examined it is obvious that daytime evaporative enrichment can only be adequately described when the transpiration-dependent mixing of ^18^O enriched water with nonenriched xylem water is taken into account. Thus Δ^18^O_es_ overestimated the actual values of Δ^18^O_L_ during the day, when transpiration rates are high (Flanagan *et al*., 1993; Wang *et al*., 1998). Moreover, night-time values can only be represented by the Péclet model when nonsteady-state conditions are also considered, a finding that is in good agreement with recent observations (Cernusak *et al*., 2005; Farquhar & Cernusak, 2005; Cuntz *et al*., 2007). The calculated scaled effective path length (L) for water movement from the veins to the site of evaporation for the four different species is well within the range reported in the literature (0.004–0.17 m from Wang *et al*., 1998; Table 3). Slight differences among values of L observed here and from literature data of the same or related species are expected as we should assume that L is not constant for a given species but affected by environmental conditions such as water relations (Ferrio *et al*., 2009, 2012), related to leaf development stage and age (Barnard *et al*., 2007), and dependent on transpiration rate (Song *et al*., 2013).

**Table:** The calculated effective path length L determined in this study compared to values of the same or of related species obtained from the literature

Species examined here	L (m)	Same or related species from literature	L (m)	Reference
*Pinus sylvestris*	0.021	*P. sylvestris*	0.05	Barnard *et al*. (2007)
		*Pinus edulis* and *P*. *monophylla*	0.008	Pendall *et al*. (2005)
*Fagus sylvatica*	0.017	*F. sylvatica*	0.017–0.052^1^	Ferrio *et al*. (2009)
		*F. sylvatica*	0.015^2^	Keitel *et al*. (2006)
*Eucalyptus delegatensis*	0.025	Different eucalypt species	0.005–0.22	Kahmen *et al*. (2008)
*Quercus petraea*	0.06	No oak species given in literature		

1 Depending on water availability; lower values with optimum water supply, high values under drought conditions.

2 At several sites across Europe.

### While the leaf water-soluble OM shows the expected ^18^O enrichment in all species examined, phloem sugars do not match predictions in some

When comparing mean daily Δ^18^O_L_ (weighted for photosynthesis or photosynthetic active radiation) with leaf water-soluble OM (Table 2), the enrichment of organic oxygen above water is very close to the expected 27‰ for the three broadleaf species and slightly lower for pine and larch, but still within the range reported for recent assimilates in other species. In *Ricinus communis,* for example, the enrichment of recent assimilates above leaf water can vary between 25.4 and 27.1‰ (Barbour *et al*., 2000; Cernusak *et al*., 2003; Gessler *et al*., 2007b). Leaf water-soluble OM is a mixture of mainly sugars, organic acids and amino acids. Slight deviations in the oxygen isotope enrichment from the average 27‰ might be attributed to compounds containing not only carbonyl oxygen, but also oxygen in other functional groups, which might differ in their isotopic composition. Whereas Δ^18^O was comparable between leaf water-soluble and phloem OM in beech and oak, phloem OM was depleted by *c*. 2‰ in pine and by < 4‰ in Alpine ash and larch. As a consequence, ^18^O enrichment of phloem OM above water was clearly lower than the expected 27‰ (Table 2). Figure 3 indicates that leaf water isotopic enrichment was more variable during the diel course than was Δ^18^O in the phloem. The damped temporal variation of phloem OM points to a longer integration of the evaporative conditions imprinted in the oxygen isotope enrichment due to longer turnover times of this OM pool. Barnard *et al*. (2007) calculated approximate turnover times between 6 and 12 h for soluble OM in pine needles, whereas leaf water turned over in *c*. 1 h during the light period. Moreover, it has been shown for various species that leaf water can turn over and reach steady-state values within almost 35 min (Wang & Yakir, 1995). Due to the time needed for phloem loading and transport, the isotopic signal arrives in the twig phloem with some additional delay. Brandes *et al*. (2006) observed that the isotopic time lag between leaf water-soluble and twig phloem OM in Scots pine was < 1 d. Together, the longer turnover time and the time needed for transport might indicate one of two hypotheses (or both): (1) that evaporative conditions and thus leaf water enrichment of the preceding day(s) affect phloem Δ^18^O and (2) that not only OM assimilated during the day, but also OM originating from transitory starch remobilized during the night contribute to phloem-transported compounds. Both factors could be partially responsible for the lower-than-expected ^18^O enrichment of phloem OM above leaf water in the two conifers and *E. delegatensis*. However, we can rule out (1) for pine and Alpine ash because the Δ^18^O of phloem OM was also clearly less enriched than the expected 27‰ when compared with photosynthesis-weighted modelled Δ^18^O_Ls_ of the preceding 2 d. Hypothesis (2) is based on the following assumption: during starch hydrolysis *c*. 6 out of 11 oxygen atoms of the produced sucrose are exchanged with the normally less enriched leaf water during the night (Gessler *et al*., 2007b). In our study, however, the average night-time Δ^18^O_L_ was comparable with photosynthesis-weighted daytime values in both pine and Alpine ash (cf. Fig. 3). As a consequence, the contribution of starch-derived organic compounds could not explain the lower-than-expected Δ^18^O values for phloem OM.

### Differences in the chemical composition of phloem OM are most likely not the reason for the lower-than-expected oxygen isotope enrichment

One reason for the relative depletion of the conifer phloem might be related to the chemical composition of organic compounds transported, as it is known that different compound classes differ in their oxygen isotope composition (Schmidt *et al*., 2001) and in their ability to exchange organic oxygen with water. The comparison between the phloem metabolome (weighted for oxygen atoms) of beech, oak and pine revealed that the most abundant compound was sucrose in all species and that the monosaccharides glucose and fructose only played a minor role. Glucose and fructose both have a free carbonyl group and could exchange one oxygen atom per molecule with phloem water. If we assume full exchange of each carbonyl oxygen of the monosaccharides with nonenriched source water, then this would cause a ^18^O depletion of phloem OM by *c*. 0.5‰; a value too small to explain the lower-than-expected ^18^O enrichment of pine phloem OM. The most obvious difference between beech, on the one hand, and oak and pine, on the other, is the relatively high contribution of cyclitols to organic oxygen in the phloem of the two latter species. To our knowledge, there is no information published on the compound-specific oxygen isotope composition of cyclitols, even though it is known that alcohols can be slightly ^18^O depleted compared to carbohydrates (Schmidt *et al*., 2001). If we were to assume that only pinitol, which contributes 5.6% to the organic oxygen, was responsible for the deviation of phloem OM from the expected 27‰ enrichment in pine, this compound would need to have an unrealistic Δ^18^O value of −35.5‰ (i.e. (23.5‰ – 0.944 × 27‰)/0.056). Moreover, in that case only pinitol and neither the precursor myo-inisitol (Kindl *et al*., 1966) present in beech and oak nor the other cyclitol quercitol should be strongly depleted. As a consequence, we can rule out differences in the chemical composition as being responsible for the observed differences in the ^18^O enrichment of phloem OM between species – at least based on the comparison between pine, on the one hand, and beech and oak, on the other.

### Phloem loading and transport as well as bark photosynthesis might explain the ^18^O enrichment patterns in the phloem

The clear and significant difference in Δ^18^O between leaf water-soluble and phloem OM in the two coniferous species and in *E. delegatensis* might be best explained by exchange of organic oxygen with water less enriched than leaf water associated with (1) phloem loading and/or (2) phloem transport together with a contribution of assimilates originating from bark photosynthesis to phloem OM.

#### Phloem loading

In contrast to broadleaf species where phloem loading occurs via the companion cells to the phloem in the minor veins which are located in close vicinity to the mesophyll cells (Lalonde *et al*., 2003), the phloem in the needles of conifers is loaded in the central cylinder, isolated by an endodermis from the needle mesophyll. The symplastic pre-phloem pathway for assimilate transport from the mesophyll cells crosses the bundle sheath, the transfusion parenchyma as well as the Strasburger cells before the sieve elements are reached (Liesche *et al*., 2011). If we now hypothesize that oxygen atom exchange occurs before or during phloem loading, explaining the low ^18^O enrichment of phloem sugars in the two conifers, the following would be prerequisites: the endodermis with the Casparian strips prevents, at least partially, the back-diffusion of enriched mesophyll water to the perfusion tissue so that at least the region directly around the axial phloem is mainly influenced by nonenriched xylem water. During the transport through the cells of the perfusion parenchyma and/or the Strasburger cells considerable amounts of the transport sugar sucrose need to be converted to substances with a free carbonyl group in order to be able to exchange oxygen atoms with the nonenriched water. A calculation with several simplifications illustrates the order of magnitude of the effect on Δ^18^O of the OM loaded into the phloem: we take into account only sucrose transported to the perfusion tissue where we assume it to be broken down to fructose and glucose; alternatively hexoses might be transported from the mesophyll to the sieve tubes. Glucose and fructose can exchange 1 oxygen atom per molecule and we assume fast equilibration between the two molecules so that 2 oxygen atoms can be exchanged per hexose. If sucrose is synthesized from hexoses within the central cylinder and loaded into the phloem, up to 3 of 11 oxygen atoms could have exchanged. We now bravely assume that the water with which the organic oxygen is exchanged is not evaporatively enriched at all (i.e. equalling the source water). Then the sucrose molecule in the phloem in pine would have a Δ^18^O of 8/11 × (11.4‰ + 27‰) + 3/11 × 27‰ = 35.3‰ when all exchangeable oxygen atoms had actually exchanged. (The photosynthesis-weighted Δ^18^O_L_ in pine is 11.4‰ and 27‰ is the expected enrichment of carbonyl groups above water.) This calculated value is comparable with the measured Δ^18^O of bulk phloem OM of 35.1‰. The same calculation for larch results in values between 34.0‰ (measured 31.7‰) and 38.9‰ (measured 33.4‰). Our calculation shows that such a mechanism could in principle explain the Δ^18^O values in the pine but not in the larch phloem. It is, however, unlikely that the water for exchange in the needle central cylinder is only influenced by xylem and not by the ^18^O-enriched leaf water. If the needle endodermis were such an effective barrier against back diffusion of mesophyll water, causing the whole central portion of the needle water to be fully xylem-like, this would also affect our calculation of lamina needle water ^18^O enrichment (Eqn 2) computed from measured bulk needle water. If *φ*_*x*_ were increased and thus *φ*_L_ decreased, the calculated lamina mesophyll water enrichment would increase. An increase of *φ*_*x*_ to 20% would cause calculated photosynthesis-weighted Δ^18^O_L_ in pine to increase by 2.5‰ and thus the Δ^18^O of phloem sucrose – as calculated above assuming the exchange of 3/11 oxygen atoms – would be 37.1‰; that is, clearly above the observed value.

In conclusion such oxygen atom exchanges processes could contribute to the observed decrease in Δ^18^O from leaf sugars to phloem OM in the two conifers to some extent but most likely cannot fully explain their lower than expected relative enrichment of OM in the phloem. Moreover the mechanism depicted above does not explain our observations in Alpine ash.

#### Phloem transport

Offermann *et al*. (2011) proposed that the widely accepted theory of phloem transport (as recently reviewed by Van Bel, 2003) can offer an explanation for exchange of organic oxygen atoms with stem water. According to theory, phloem transport is associated with continuous unloading and reloading of sugars along the transport path. It has been shown that up to 6% of the phloem sugars are unloaded per cm of transport path and that two-thirds of these carbohydrates are reloaded into the sieve tubes again (Minchin & Thorpe, 1987). If we assume that part of the retrieved sugars underwent metabolic conversion in the parenchymatic tissues of the twig or trunk forming intermediates with exchangeable carbonyl groups, we could then explain the ^18^O depletion of phloem OM compared to leaf sugars. Moreover, bark photosynthesis and phloem loading of bark assimilated sugars could contribute to the lower-than-expected ^18^O enrichment in the phloem. Organic matter fixed (or refixed from respired CO_2_) in the bark, where reaction water is not or only slightly enriched, should have a Δ^18^O value of *c*. 27‰ above source water (Cernusak *et al*., 2005), well below the enrichment for sugars fixed in leaves and the values observed in phloem OM of the two conifers and Alpine ash. Thus bark photosynthesis, which mainly occurs in young twigs, could contribute to the twig phloem OM of pine, larch and ash trees and explain the observed Δ^18^O values. In fact, Berveiller *et al*. (2007) observed higher maximum gross photosynthetic rates under light-saturated conditions for stem photosynthesis for pine (2.9 μmol m^−2^ s^−1^) than for beech and oak. There is no information on the photosynthetic capacity of the bark of *E*. *delegatensis*, but Tausz *et al*. (2005) found that the sun-exposed bark of *Eucalyptus nitens* has the photochemical properties and the pigment composition of a sun leaf. Tausz *et al*. (2005) conclude that twig and stem CO_2_ fixation is thus likely to be important in smooth-barked eucalypts (such as *E. delegatensis*), because the leaf area index of eucalypt forests is low compared to European beech or oak forests, and eucalypt leaves are pendulous. The same is true for the two conifers examined, which have very sparse canopies and low LAI (Brandes *et al*., 2007) and thus self-shading of the bark by leaves is lower than for the two broadleaf species. As a consequence, it is probable that actual bark photosynthesis is higher in the two conifers and in Alpine ash than in beech and oak, thus contributing to an explanation for the observed lower oxygen enrichment in the phloem-transported sugars.

In conclusion, we demonstrate here that even though leaf water enrichment seems to be imprinted on leaf assimilates as expected, the phloem-transported OM enrichment strongly differs between the two deciduous broadleaf species, on the one hand, and the two conifers and the evergreen broadleaf species, on the other. We have presented two mechanisms related to (1) phloem loading in the conifers and to (2) phloem transport and bark photosynthesis, that could explain the lower-than-expected evaporative enrichment of phloem OM in three out of five species examined. Both mechanisms cause a decoupling of the original leaf-level isotope signal, thus affecting the interpretation of the oxygen isotopic signature in heterotrophic tissues including tree rings. The findings in our study point to the possibility that oxygen atom exchange with xylem water might occur not only during cellulose synthesis in the stem, but also – at least in some species – before the sugars arrive in the cambial tissues. In the conifers and the eucalypt species the ‘oxygen atom exchange’ (assuming the exchanged water equals source water) calculated for phloem sugars ranges between 30% (pine) and 63% (larch treeline). It should be noted that the values we calculate here are not necessarily exchange *sensu strictu*, but also include mixing of sugars produced in a nonenriched aqueous environment. Nevertheless, the values describe the decoupling between leaf water enrichment and the Δ^18^O of OM. The values are comparable to or even higher than the average exchange of 42% assumed for cellulose synthesis from sucrose (Cernusak *et al*., 2005), and need to be taken into account when studying tree ring δ^18^O. Figure 4(c) (pine) might imply that the phloem OM values only show an offset compared to leaf assimilates, but the results from larch (Fig. 4e) suggest that the difference might not be constant, and may depend on environmental conditions and phenology. Our results demonstrate that we need to obtain more information about the mechanisms that control the transfer of the oxygen isotopic signal from the leaf to heterotrophic tissues. This is of particular importance for tree ring-based climate reconstructions and for studies that explore the oxygen isotope signature of the tree ring archive to study plant–climate interactions. Without knowledge of the mechanisms that uncouple the cellulose oxygen isotope signal from leaf level signals and the quantitative extent of this uncoupling, the interpretation of the tree ring oxygen isotope composition will remain error-prone.

## Supporting Information

**Notes S1** Evaporative enrichment models: steady-state isotopic enrichment of ^18^O over source water at the site of evaporation in the leaf (Δ^18^O_es_) under steady-state conditions, steady-state isotopic enrichment of mean lamina mesophyll water (Δ^18^O_Ls_) taking into account the the *P’eclet* effect and nonsteady-state enrichment of mean lamina mesophyll water above source water (Δ^18^O_Ln_).

## References

[b1] Barbour MM (2007). Stable oxygen isotope composition of plant tissue: a review. Functional Plant Biology.

[b2] Barbour MM, Farquhar GD (2000). Relative humidity- and ABA-induced variation in carbon and oxygen isotope ratios of cotton leaves. Plant, Cell & Environment.

[b3] Barbour MM, Roden JS, Farquhar GD, Ehleringer JR (2004). Expressing leaf water and cellulose oxygen isotope ratios as enrichment above source water reveals evidence of a Peclet effect. Oecologia.

[b4] Barbour MM, Schurr U, Henry BK, Wong SC, Farquhar GD (2000). Variation in the oxygen isotope ratio of phloem sap sucrose from castor bean. Evidence in support of the Peclet effect. Plant Physiology.

[b5] Barbour MM, Walcroft AS, Farquhar GD (2002). Seasonal variation in delta ^13^C and delta ^18^O of cellulose from growth rings of *Pinus radiata*. Plant, Cell & Environment.

[b6] Barnard RL, Salmon Y, Kodama N, Sorgel K, Holst J, Rennenberg H, Gessler A, Buchmann N (2007). Evaporative enrichment and time lags between delta ^18^O of leaf water and organic pools in a pine stand. Plant, Cell & Environment.

[b7] Beadle CL, Neilson RE, Talbot H, Jarvis PG (1985). Stomatal conductance and photosynthesis in a mature Scots pine forest.1. Diurnal, seasonal and spatial variation in shoots. Journal of Applied Ecology.

[b8] van Bel AJE, Hess PH (2008). Hexoses as phloem transport sugars: the end of a dogma?. Journal of Experimental Botany.

[b9] Berveiller D, Kierzkowski D, Damesin C (2007). Interspecific variability of stem photosynthesis among tree species. Tree Physiology.

[b10] Brandes E, Kodama N, Whittaker K, Weston C, Rennenberg H, Keitel C, Adams MA, Gessler A (2006). Short-term variation in the isotopic composition of organic matter allocated from the leaves to the stem of *Pinus sylvestris*: effects of photosynthetic and postphotosynthetic carbon isotope fractionation. Global Change Biology.

[b11] Brandes E, Wenninger J, Koeniger P, Schindler D, Rennenberg H, Leibundgut C, Mayer H, Gessler A (2007). Assessing environmental and physiological controls over water relations in a Scots pine (*Pinus sylvestris* L.) stand through analyses of stable isotope composition of water and organic matter. Plant, Cell & Environment.

[b12] von Caemmerer S, Farquhar GD (1981). Some relationships between the biochemistry of photosynthesis and the gas-exchange of leaves. Planta.

[b13] Cernusak LA, Farquhar GD, Pate JS (2005). Environmental and physiological controls over oxygen and carbon isotope composition of Tasmanian blue gum, *Eucalyptus globulus*. Tree Physiology.

[b14] Cernusak LA, Wong SC, Farquhar GD (2003). Oxygen isotope composition of phloem sap in relation to leaf water in *Ricinus communis*. Functional Plant Biology.

[b15] Craig H, Gordon LI, Tongiorgi E (1965). Deuterium and oxygen-18 variations in the ocean and the marine atmosphere. Proceedings of a conference on stable isotopes in oceanographic studies and palaeotemperatures.

[b16] Cuntz M, Ogee J, Farquhar GD, Peylin P, Cernusak LA (2007). Modelling advection and diffusion of water isotopologues in leaves. Plant, Cell & Environment.

[b17] DeNiro MJ, Epstein S (1979). Relationship between the oxygen isotope ratios of terrestrial plant cellulose, carbon dioxide, and water. Science.

[b18] DeNiro MJ, Epstein S (1981). Isotopic composition of cellulose from aquatic organisms. Geochimica et Cosmochimica Acta.

[b19] Dongmann G, Nürnberg HW, Förstel H, Wagener K (1974). On the enrichment of H_2_^18^O in the leaves of transpiring plants. Radiation and Environmental Biophysics.

[b20] Ehleringer JR, Roden JS, Dawson T, Sala OE, Jackson R, Mooney HA, Howarth R (2000). Assessing ecosystem-level water relations through stable isotope ratio analyses. Methods in ecosystem science.

[b21] Erxleben A, Gessler A, Vervliet-Scheebaum M, Reski R (2011). Metabolite profiling of the moss *Physcomitrella patens* reveals evolutionary conservation of osmoprotective substances. Plant Cell Reports.

[b22] Farquhar GD, Barbour MM, Henry BK, Griffiths H (1998). Interpretation of oxygen isotope composition of leaf material. Chapter 3, Stable isotopes: integration of biological, ecological and geochemical processes.

[b23] Farquhar GD, Cernusak LA (2005). On the isotopic composition of leaf water in the non-steady state. Functional Plant Biology.

[b24] Farquhar GD, Gan KS (2003). On the progressive enrichment of the oxygen isotopic composition of water along a leaf (Reprinted from Plant, Cell and Environment, vol 26, pg 801–819, 2003). Plant, Cell & Environment.

[b26] Farquhar GD, Lloyd J, Ehleringer JR, Hall AE, Farquhar GD (1993). Carbon and oxygen isotope effects in the exchange of carbon dioxide between terrestrial plants and the atmosphere. Stable isotopes and plant carbon–water relations.

[b27] Ferrio JP, Cuntz M, Offermann C, Siegwolf R, Saurer M, Gessler A (2009). Effect of water availability on leaf water isotopic enrichment in beech seedlings shows limitations of current fractionation models. Plant, Cell & Environment.

[b28] Ferrio JP, Pou A, Florez-Sarasa I, Gessler A, Kodama N, Flexas J, Ribas-Carbo M (2012). The Péclet effect on leaf water enrichment correlates with leaf hydraulic conductance and mesophyll conductance for CO_2_. Plant, Cell & Environment.

[b29] Flanagan LB, Marshall JD, Ehleringer JR (1993). Photosynthetic gas-exchange and the stable-isotope composition of leaf water – comparison of a xylem-tapping mistletoe and its host. Plant, Cell & Environment.

[b30] Gessler A, Brandes E, Buchmann N, Helle G, Rennenberg H, Barnard R (2009a). Tracing carbon and oxygen isotope signals from newly assimilated sugars in the leaves to the tree ring archive. Plant, Cell & Environment.

[b31] Gessler A, Keitel C, Kodama N, Weston C, Winters AJ, Keith H, Grice K, Leuning R, Fraquhar GD (2007a). δ^13^C of organic matter transported from the leaves to the roots in *Eucalyptus delegatensis*: short-term variations and relation to respired CO_2_. Functional Plant Biology.

[b32] Gessler A, Peuke AD, Keitel C, Farquhar GD (2007b). Oxygen isotope enrichment of organic matter in *Ricinus communis* during the diel course and as affected by assimilate transport. New Phytologist.

[b33] Gessler A, Rennenberg H, Keitel C (2004). Stable isotope composition of organic compounds transported in the phloem of European beech – evaluation of different methods of phloem sap collection and assessment of gradients in carbon isotope composition during leaf-to-stem transport. Plant Biology.

[b34] Gessler A, Tcherkez G, Karyanto O, Keitel C, Ferrio JP, Ghashghaie J, Kreuzwieser J, Farquhar GD (2009b). On the metabolic origin of the carbon isotope composition of CO_2_ evolved from darkened light-adapted leaves in *Ricinus communis*. New Phytologist.

[b35] Grams TEE, Kozovits AR, Haberle KH, Matyssek R, Dawson TE (2007). Combining delta ^13^C and delta ^18^O analyses to unravel competition, CO_2_ and O_3_ effects on the physiological performance of different-aged trees. Plant, Cell & Environment.

[b36] Granier A, Biron P, Lemoine D (2000). Water balance, transpiration and canopy conductance in two beech stands. Agricultural and Forest Meteorology.

[b37] Helliker BR, Ehleringer JR (2002). Differential ^18^O enrichment of leaf cellulose in C_3_ versus C_4_ grasses. Functional Plant Biology.

[b38] Holst J, Barnard R, Brandes E, Buchmann N, Gessler A, Jaeger L (2008). Impacts of summer water limitation on the carbon balance of a Scots pine forest in the southern upper Rhine plane. Agricultural & Forest Meteorology.

[b39] Kahmen A, Simonin K, Tu K, Merchant A, Callister A, Siegwolf R, Dawson T, Arndt S (2008). Effects of environmental parameters, leaf physiological properties and leaf water relations on leaf water delta ^18^O enrichment in different *Eucalyptus* species. Plant, Cell & Environment.

[b40] Keitel C, Matzarakis A, Rennenberg H, Gessler A (2006). Carbon isotopic composition and oxygen isotopic enrichment in phloem and total leaf organic matter of European beech (*Fagus sylvatica* L.) along a climate gradient. Plant, Cell & Environment.

[b41] Kindl H, Scholda R, Hoffmann-Ostenhof O (1966). The biosynthesis of cyclitols. Angewandte Chemie-International Edition in English.

[b42] Kopka J, Schauer N, Krueger S, Birkemeyer C, Usadel B, Bergmüller E, Dörmann P, Weckwerth W, Gibon Y, Stitt M (2005). GMD @ CSB.DB: the Golm Metabolome Database. Bioinformatics.

[b43] Lalonde S, Tegeder M, Throne-Holst M, Frommer W, Patrick J (2003). Phloem loading and unloading of sugars and amino acids. Plant, Cell & Environment.

[b44] Leuning R, Cleugh HA, Zegelin SJ, Hughes D (2005). Carbon and water fluxes over a temperate Eucalyptus forest and a tropical wet/dry savanna in Australia: measurements and comparison with MODros. Inf. Serv. remote sensing estimates. Agricultural and Forest Meteorology.

[b45] Liesche J, Martens HJ, Schulz A (2011). Symplasmic transport and phloem loading in gymnosperm leaves. Protoplasma.

[b46] Lin J, Jach ME, Ceulemans R (2001). Stomatal density and needle anatomy of Scots pine (*Pinus sylvestris*) are affected by elevated CO_2_. New Phytologist.

[b47] McDowell N, Pockman WT, Allen CD, Breshears DD, Cobb N, Kolb T, Plaut J, Sperry J, West A, Williams DG (2008). Mechanisms of plant survival and mortality during drought: why do some plants survive while others succumb to drought?. New Phytologist.

[b48] Minchin P, Thorpe M (1987). Measurement of unloading and reloading of photo-assimilate within the stem of bean. Journal of Experimental Botany.

[b49] Moser L, Fonti P, Büntgen U, Franzen J, Esper J, Luterbacher J, Frank D (2010). Timing and duration of European larch growing season along altitudinal gradients in the Swiss Alps. Tree Physiology.

[b50] Offermann C, Ferrio JP, Holst J, Grote R, Siegwolf R, Kayler Z, Gessler A (2011). The long way down-are carbon and oxygen isotope signals in the tree ring uncoupled from canopy physiological processes?. Tree Physiology.

[b51] Pendall E, Williams DG, Leavitt SW (2005). Comparison of measured and modeled variations in pinon pine leaf water isotopic enrichment across a summer moisture gradient. Oecologia.

[b52] Richter A, Wanek W, Werner RA, Ghashghaie J, Jaggi M, Gessler A, Brugnoli E, Hettmann E, Gottlicher SG, Salmon Y (2009). Preparation of starch and soluble sugars of plant material for the analysis of carbon isotope composition: a comparison of methods. Rapid Communications in Mass Spectrometry.

[b53] Roden JS, Bowling DR, McDowell NG, Bond BJ, Ehleringer JR (2005). Carbon and oxygen isotope ratios of tree ring cellulose along a precipitation transect in Oregon, United States. Journal of Geophysical Research-Biogeosciences.

[b54] Roden JS, Ehleringer JR (1999a). Hydrogen and oxygen isotope ratios of tree-ring cellulose for riparian trees grown long-term under hydroponically controlled environments. Oecologia.

[b55] Roden JS, Ehleringer JR (1999b). Observations of hydrogen and oxygen isotopes in leaf water confirm the Craig-Gordon model under wide-ranging environmental conditions. Plant Physiology.

[b56] Roden JS, Lin GG, Ehleringer JR (2000). A mechanistic model for interpretation of hydrogen and oxygen isotope ratios in tree-ring cellulose. Geochimica et Cosmochimica Acta.

[b57] Saurer M, Schweingruber F, Vaganov EA, Shiyatov SG, Siegwolf R (2002). Spatial and temporal oxygen isotope trends at the northern tree-line in Eurasia. Geophysical Research Letters.

[b58] Schmidt HL, Werner RA, Rossmann A (2001). ^18^O pattern and biosynthesis of natural plant products. Phytochemistry.

[b59] Schneider S, Gessler A, Weber P, von Sengbusch D, Hanemann U, Rennenberg H (1996). Soluble N compounds in trees exposed to high loads of N: a comparison of spruce (*Picea abies*) and beech (*Fagus sylvatica*) grown under field conditions. New Phytologist.

[b60] Song X, Barbour MM, Farquhar GD, Vann DR, Helliker BR (2013). Transpiration rate relates to within- and across- species variations in effective pathlength in a leaf water model of oxygen isotope enrichment. Plant, Cell & Environment.

[b61] Sternberg L (2009). Oxygen stable isotope ratios of tree-ring cellulose: the next phase of understanding. New Phytologist.

[b62] Sternberg L, Deniro MJ (1983). Bio-geochemical implications of the isotopic equilibrium fractionation factor between oxygen atoms of acetone and water. Geochimica et Cosmochimica Acta.

[b63] Sternberg L, Ellsworth PFV (2011). Divergent biochemical fractionation, not convergent temperature, explains cellulose oxygen isotope enrichment across latitudes. PLoS ONE.

[b64] Sternberg LDL, Deniro MJ, Savidge RA (1986). Oxygen isotope exchange between metabolites and water during biochemical reactions leading to cellulose synthesis. Plant Physiology.

[b65] Takemoto Y, Greenwood MS (1993). Maturation in larch: age-related changes in xylem development in the long-shoot foliage and the main stem. Tree Physiology.

[b66] Tausz M, Warren C, Adams M (2005). Is the bark of shining gum (*Eucalyptus nitens*) a sun or a shade leaf?. Trees-Structure and Function.

[b67] Treydte KS, Schleser GH, Helle G, Frank DC, Winiger M, Haug GH, Esper J (2006). The twentieth century was the wettest period in northern Pakistan over the past millennium. Nature.

[b68] Van Bel AJE (1993). Strategies of phloem loading. Annual Review of Plant Physiology and Plant Molecular Biology.

[b69] Van Bel AJE (2003). The phloem, a miracle of ingenuity. Plant, Cell & Environment.

[b70] Van Bel AJE, Gamalei YV (1992). Ecophysiology of phloem loading in source leaves. Plant, Cell & Environment.

[b71] Walcroft AS, Silverster WB, Whitehead D, Kelliher FM (1997). Seasonal changes in stable carbon isotope ratios within annual rings of *Pinus radiata* reflect environmental regulation of grwoth processes. Australian Journal of Plant Physiology.

[b72] Wang XF, Yakir D (1995). Temporal and spatial variations in the oxygen-18 content of leaf water in different plant species. Plant, Cell & Environment.

[b73] Wang XF, Yakir D, Avishai M (1998). Non-climatic variations in the oxygen isotopic compositions of plants. Global Change Biology.

[b74] Werner C, Schnyder H, Cuntz M, Keitel C, Zeeman MJ, Dawson TE, Badeck FW, Brugnoli E, Ghashghaie J, Grams TEE (2012). Progress and challenges in using stable isotopes to trace plant carbon and water relations across scales. Biogeosciences.

[b75] Yakir D, Deniro MJ (1990). Oxygen and hydrogen isotope fractionation during cellulose metabolism in *Lemna gibba* L. Plant Physiology.

