# Supplementary material for: The oxygen isotope enrichment of leaf-exported assimilates – does it always reflect lamina leaf water enrichment?
Source: New Phytol. 2013 Jun 13;200(1):144–57. doi: 10.1111/nph.12359 (PMC3902987; doi:10.1111/nph.12359)
Supplement: Supplementary file 1 — Notes S1 Evaporative enrichment models: steady-state isotopic enrichment of 18O over source water at the site of evaporation in the leaf (Δ18Oes) under steady-state conditions, steady-state isotopic enrichment of mean lamina mesophyll water (Δ18OLs) taking into account the the P’eclet effect and nonsteady-state enrichment of mean lamina mesophyll water above source water (Δ18OLn). [file nph0200-0144-sd1.docx]

**Supporting Information Notes S1 Evaporative enrichment models**

Steady-state isotopic enrichment of ^18^O over source water at the site of evaporation in the leaf (Δ^18^O*_es_*) under steady state conditions was calculated as follows ([Craig & Gordon, 1965](#_ENREF_16); [Dongmann *et al.*, 1974](#_ENREF_20)):

__  Eqn S1

where *ε^+^* is the equilibrium fractionation between liquid water and water vapour; *ε_k_* is the kinetic fractionation as vapour diffuses from leaf intercellular spaces to the atmosphere ([Farquhar *et al.*, 1989](#_ENREF_25)), Δ^18^O*_v_* is the isotopic enrichment of water vapour relative to the source water taken up by the plant (i.e. xylem water), and *e_a_*/*e_i_* is the ratio of ambient to intercellular vapour pressures. *ε^+^* was calculated following [Bottinga and Craig (1969](#_ENREF_9)) and *ε_k_* was estimated following [Farquhar *et al.* (1989](#_ENREF_25)) taking into account the fractionation factor given by [Cappa *et al.* (2002](#_ENREF_12)). Average lamina mesophyll water is, however, expected to be less enriched than the water at the evaporative sites, resulting in an isotopic gradient between the leaf vein and the evaporative sites. The steady-state isotopic enrichment of mean lamina mesophyll water (Δ^18^O*_Ls_*) can be described by correcting Eqn S1 for the *Péclet* effect ([Farquhar & Lloyd, 1993](#_ENREF_26)), as shown in Eqn S2. The *Péclet* effect is the net effect of the convection of unenriched source water to the leaf evaporative sites via the transpiration stream as opposed by the diffusion of evaporatively enriched water away from the sites of evaporation.

 with Eqn S2

℘ is the *Péclet* number, *E* the leaf transpiration rate (mol m^-2^ s^-1^), *L* is the scaled effective path length (m) for water movement from the xylem to the site of evaporation, *C* the molar concentration of water (mol m^-3^), and *D* the diffusivity (m^2^ s^-1^) of the H_2_^18^O isotopologue in ‘normal’ water. The scaled effective path length was estimated by fitting the non-steady state model to the measured Δ^18^O of leaf water (Δ^18^O_L_) under expected steady state conditions, that typically occur at the end of the afternoon.

Under non steady-state conditions, the enrichment of mean lamina mesophyll water above source water (Δ^18^O_Ln_) can be calculated following [Farquhar & Cernusak, 2005](#_ENREF_23):

 Eqn S3

where *α^+^*=1+(*ε^+^*) and *α_k_*=1+(*ε_k_*), *W* is the lamina leaf water concentration (mol m^-2^), *t* is time (s), *g* is the total conductance to water vapour of stomata and boundary layer (mol m^-2^ s^-1^), and *w_i_* is the mole fraction of water vapour in the leaf intercellular air spaces (mol mol^-1^). *W* was estimated based on the bulk leaf water content (for pine and Alpine ash corrected for the proportion of vascular water) and leaf area measurements. The Péclet number used in the non-steady state model was estimated with the steady state model. The equation has an analytical solution that was calculated with the ‘Solver’ function in Excel. The model requires initial values for Δ^18^O_LN_ and *W* for a time point (t_0_-1) preceding the first observation. To initialise the model we took the values from the next day at the measurement time point preceding the starting time.

**References**

**Craig H, Gordon LI 1965.** Deuterium and oxygen-18 variations in the ocean and the marine atmosphere. In: Tongiorgi E, ed. *Proceedings of a Conference on Stable Isotopes in Oceanographic Studies and Palaeotemperatures*. Spoleto, Italy: Lischi and Figli, 9–130.

**Dongmann G, Nürnberg HW, Förstel H, Wagener K. 1974.** On the enrichment of H_2_^18^O in the leaves of transpiring plants. *Radiation and Environmental Biophysics* **11**: 41–52.

**Farquhar GD, Hubick KT, Condon AG, Richards RA 1989.** Carbon isotope discrimination and water-use efficiency. In: Rundel PW, Ehleringer JR, Nagy KA, eds. *Stable isotopes in ecological research*. New York, USA: Springer, 21–46.

**Bottinga Y, Craig H. 1969.** Oxygen isotope fractionation between CO_2_ and water, and the isotopic composition of marine atmospheric CO_2_. *Earth and Planetary Science Letters* **5**: 285–295.

**Cappa CD, Hendricks MB, DePaolo DJ, Cohen RC. 2002.** Isotopic fractionation of water during evaporation. *Journal of Geophysical Research-Atmosphere* **108**: D16.

**Farquhar GD, Lloyd J 1993.** Carbon and oxygen isotope effects in the exchange of carbon dioxide between terrestrial plants and the atmosphere. In: Ehleringer JR, Hall AE, Farquhar GD, eds. *Stable isotopes and plant carbon–water relations*. San Diego, CA, USA: Academic Press, 47–70.

**Farquhar GD, Cernusak LA. 2005.** On the isotopic composition of leaf water in the non-steady state. *Functional Plant Biology* **32**: 293–303.
